# Volatile Metabolite Profiles of Robusta Green Bean Coffee From Different Geographical Origins in West Java and Their Correlation With Temperature, Rainfall, and Altitudes Using SPME GC-MS–Based Metabolomics

**DOI:** 10.1155/2024/6908059

**Published:** 2024-10-26

**Authors:** Erly Marwani, Tati Surjati Syamsudin, Suci Awaliyah, Rijanti Rahaju Maulani, Asep Hidayat, Ujang Dinar Husyari, Srinanan Widiyanto

**Affiliations:** School of Life Sciences and Technology, Institut Teknologi Bandung, Bandung, Indonesia

## Abstract

The chemical composition, including volatile metabolites of green coffee beans, is influenced by geographic origin. The aim of this study was to reveal the volatile metabolite profile of a single variety of Robusta green bean coffee from five major plantation regions in West Java and to correlate these profiles with temperature, rainfall, and altitude. By using solid phase micro extractions and gas chromatography-mass spectrometry, 143 different volatile compounds were detected, with aromatic hydrocarbon, alcohols, monoterpene, pyrazines, sesquiterpenes, carboxylic acids, and terpene the most dominant. Principal component analysis (PCA) indicated 64.3% variability, showing that the metabolite profile of Robusta green coffee from the Bogor region was distinctly different from those in Ciamis, Kuningan, Sumedang, and Tasikmalaya, which were more similar to each other. Metabolites such as benzaldehyde, isovaleric acid, toluene, diisobutyl succinate, 1-heptene, 4-dodecene, caffeine, acetic acid, and methyl benzoate were identified as key discriminants, with a VIP score greater than 1.5. Temperature increases were linked to higher levels of isovaleric acid, diisobutyl succinate, 4-dodecene, toluene, and acetic acid, while other discriminant metabolites declined. Increased rainfall was associated with higher levels of benzaldehyde, 1-heptene, caffeine, and methyl benzoate, but lower levels of the other discriminants. Altitude had a positive correlation with methyl benzoate and 1-heptene, and a negative correlation with isovaleric acid and 4-dodecene, with weaker correlations for other compounds. In summary, Robusta green coffee beans from different regions of West Java can be distinguished by their volatile metabolites. Bogor green coffee beans had higher levels of benzaldehyde, 1-heptene, caffeine, and methyl benzoate, Kuningan beans had more diisobutyl succinate and 4-dodecene, Ciamis beans had higher levels of isovaleric acid, diisobutyl succinate, and 4-dodecene, while Sumedang and Tasikmalaya beans were similar, with higher levels of isovaleric acid, diisobutyl succinate, 4-dodecene, toluene, and acetic acid. This difference is related to the climatic factors of temperature and rainfall, as well as the altitude at which Robusta coffee is grown.

## 1. Introduction

Coffee is one of the most popular drinks; thus, coffee trees are widely cultivated all over the world, especially trees of *Coffea arabica* and *Coffea canephora* var. Robusta. Indonesia is known as the fourth largest coffee producer in the world, after Brazil, Vietnam, and Colombia. The coffee plantation area in Indonesia in 2020 was around 1.23 million hectares which includes Arabica coffee plantations covering an area of 366,414 ha and Robusta coffee plantations covering 860,777 ha. Robusta coffee plantations in west Java reach an area of 18,642 ha which is spread over many districts and cities. The six largest areas (the main producing areas) of robusta coffee plantations in west Java are located in Bogor (6016 ha), Ciamis (2241 ha), Tasikmalaya (1996 ha), Kuningan (1675 ha), Cianjur (1044 ha), and Sumedang (907 ha). Yields from Robusta coffee plantations from West Java are generally traded in the form of green bean coffee to the domestic market and to the global international market [1].

The quality of coffee is determined by several characteristics, generally which include green bean size and defect, and the origin of coffee is produced; however, in the end, consumers judge the quality of coffee from its sensory quality, especially through its quality of aroma and flavor [2]. In addition, the coffee sensory quality is related to the metabolites that are present in green bean coffee.

Some studies indicated that coffee quality was influenced by the presence and concentrations of primary and secondary metabolites that have a role in a sensory (i.e., organoleptic) attributes, shelf stability, and nutritional aspects of coffee [3]. Similarly, coffee quality ranking can be done based on chemical analysis of key compounds attributed to quality (targeted compounds), which include biochemical compounds in the form of caffeine, chlorogenic acid, trigonelline, sucrose, and lipids [4]. However, comprehensive metabolite profiling is more useful in evaluating a food product including coffee than using conventional analysis which only evaluates certain target compounds [5, 6]. This is because the composition of the metabolites contained in green coffee beans or roasted beans is very abundant which consists of volatile and nonvolatile metabolites [7].

The overall chemical composition and metabolite content or metabolite profile in green coffee beans may vary, and this variation may be influenced by the type of coffee plant genotype [8], environmental factors such as variation from altitude, soil type, temperature, and shade where the coffee plant is grown [9–12]; seasonal variation [13]; geographic origin [14]; pre and postharvest processing of the beans, that is, wet or dry processing [15]); the presence of defective seeds [16]; and fruit maturity [17].

The overall metabolite profile data of a coffee sample has been used for various purposes, including to distinguish a coffee sample from other coffees based on genotype (Arabica coffee vs. Robusta coffee); geographical origin, the environment where coffee plants grow such as the difference in height, and the authenticity of a particular type of coffee. It also has been used for differentiating the coffee quality of Arabica and Robusta coffee as reported by [18]. Furthermore, [19] succeeded in revealing the key volatile metabolites to differentiate Arabica and Robusta coffees through an analysis of their entire volatile compounds. In addition, [20] succeeded in identifying compounds that characterize sensory quality and authenticity in ground coffee through the analysis of overall chemical compounds through a metabolomics approach.

Metabolomics is a comprehensive and quantitative analysis of all small metabolites in a biological system [21, 22] and is used to study the total metabolites in biological samples [23, 24]. It is now widely used to study biological samples of plants, microbes, animals, and humans. Furthermore, metabolomics offers high throughput and total profiling of metabolites has become increasingly important in food science and technology [22, 25]. Metabolomics workflow includes sample preparation; metabolites analysis using analytical instruments such as GC-MS, LC-MS, and ^1^H NMR; and statistical analysis for interpretation of the large data sets. Metabolomics analysis can be carried out using software such as SIMCA-P and MetaboAnalyst [26].

Studies of the total chemical composition in coffee beans have been carried out using metabolomics approaches including determining the origin of coffee [25, 27, 28] and classifying green coffee beans based on their origin and variety [28]. In addition, through overall metabolite analysis combined with metabolomics analysis [29], it could discriminate a regular coffee and a civet coffee [30], able to reveal the profile of the overall volatile chemical composition of coffee grown at different altitudes and its correlation [20], and succeeded in identifying compounds that characterize sensory quality and authenticity in ground coffee.

Flavor and aroma attributes of coffee are influenced by the set of volatile and nonvolatile metabolites that accumulate in green coffee beans. These metabolites act as precursors of the compounds contained in roasted coffee beans [31–33]. Thus, the composition of metabolites in green coffee beans forms the basis for the metabolites content in roasted coffee, for example, trigonelline in green coffee beans is degraded to pyridine after the coffee beans are roasted, as well as chlorogenic acid which undergoes a reaction to become ferulic acid after being roasted [34]. This means that the chemical composition, including volatile organic compounds, in green coffee beans plays an important role in forming the chemical composition of roasted beans, which will impact the final coffee cup profile.

The volatile compounds in biological material can be studied using the SPME-GC/MS technique, as the solid-phase microextraction (SPME) has been widely used and gives a valid technique for the analysis of headspace aroma released from food matrices, including roasted coffee beans [35–39]. Additionally, [19] states that the SPME method is nondestructive and noninvasive to the sample; thus, the sample could be used for further analysis.

In addition, [34] has conducted a study of the variability of volatile compounds in a single type of Arabica and Robusta coffee through the analysis of all volatile metabolites with SPME-GC-MS and found that the composition of volatile compounds can effectively serve as a valid indicator of the original coffee to model a reliable classification.

To the best of our knowledge, the total chemical composition of Robusta coffee beans originating from different geographic origins in West Java has not been fully documented. The chemical composition, including volatile metabolites of coffee beans, is influenced by geographic origin [4]. Geographic origin refers to the unique characteristics of each region such as soil conditions with various living organisms; complex environmental factors including altitude, temperature, rainfall, and light intensity; and the sociocultural conditions that influence coffee cultivation practices [40]. Among these geographic origin factors, temperature and rainfall significantly affect the chemical composition of the beans [41]. In the context of the recent focus on climate change issues, this study is aimed at revealing how temperature, rainfall, and altitude correlate with the metabolite profiles of Robusta green coffee beans from West Java. Using an SPME GC-MS-based metabolomics approach, the study investigates the impact of environmental factors including temperature, rainfall, and altitude on volatile metabolite profiles. Understanding these correlations can reveal how climate change affects Robusta coffee bean quality and provide valuable information to be used for monitoring the effect of climate change on Robusta coffee cultivation in the region, thereby helping farmers to develop adaptation strategies for climate change in coffee cultivation.

## 2. Materials and Methods

### 2.1. Sampling Location

**Figure 1:**
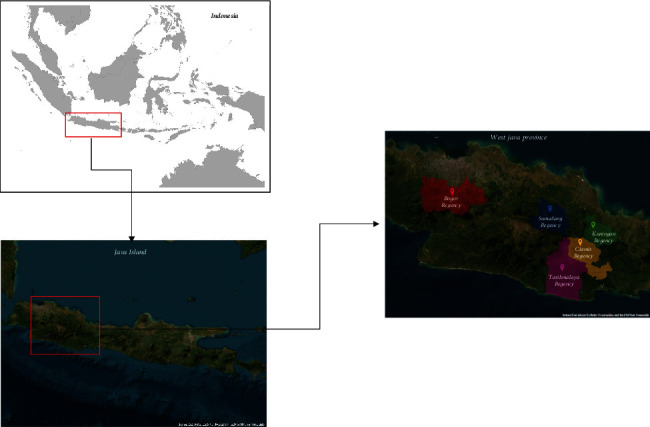
Map of West Java showing the locations of Bogor, Ciamis, Kuningan, Sumedang, and Tasikmalaya regencies.

Sampling locations were selected from the main Robusta coffee–producing regions in the province of West Java, covering an area of around 1000 ha or more, namely, Bogor (6016 ha), Ciamis (2241 ha), Kuningan (1675 ha), Sumedang (907 ha), and Tasikmalaya (1996 ha) regencies (Figure 1).

### 2.2. Sample Collection

The coffee sample used in this study was *Coffea canephora* var. Robusta, commonly referred to as Robusta coffee. Sample set of single green beans of Robusta coffee was collected from smallholder farmers at five different cultivation areas in West Java, namely, Bogor, Ciamis, Kuningan, Sumedang, and Tasikmalaya regencies (Table 1). The Robusta coffee seeds from the five locations originated from Lampung, South Sumatra. The seeds are certified by the Ministry of Agriculture of the Republic of Indonesia as Robusta coffee clone Korola [42]. Sample collection was carried out in the same harvest season in early 2021. At each location, three biological material replicates were taken resulting in a total of 15 samples of robusta green coffee beans. Care was taken to ensure that the coffee samples were sourced from plantation areas employing uniform cultivation methods, cherries of uniform maturity, consistent cherry-picking techniques, and utilizing the same postharvest process, which involved dry processing. After being dry processed by the farmers, the green beans were collected and stored at −20°C in our laboratory for further use.

### 2.3. Chemicals

Standard alkanes (C8-C23) that were used for calculating retention indices (RIs) or LRIs (linear retention indices) were purchased from Sigma–Aldrich (Santa Clara, CA, United States).

### 2.4. Extraction and SPME GC/MS-Analysis

The samples of Robusta green coffee beans from each location were ground using a coffee grinder (Cyprus grinder GR006), and 3 g of each ground green coffee bean sample was placed in a 22 mL SPME clear glass fiber vial with a PTFE/silicone septa and extracted at 70°C for 30 min using a 2-cm DVB/CAR/PDMS fiber (Supelco, Bellefonte, PA, United States). The extraction followed the method described by [19], with minor adjustments. The fiber was then subjected to thermal desorption in the GC injection port for 5 min. The GC-MS analyses were carried out using an Agilent 7890A CG system and Agilent 5975CXL EI/CI MS (Agilent Technology). The capillary column used had a specification of DB-Wax (30 m × 250 *μ*m × 0.25 *μ*m), and helium was used as the carrier gas at a flow rate of 0.8 mL/min. The column temperature was initially set to 60°C for 2 min and then increased by 5°C/min until it reached 240°C. The interface was at 280°C, and the mass scan ranged from 29 to 550 amu. The MS source temperature was 230°C, the MS quad temperature was 150°C, and the NIST14 library was utilized. The output of the GC-MS analysis consisted of spectra matched with standard compounds in the NIST 14 library. The data from the spectra, including *m*/*z*, retention time, and intensity, were tabulated in CSV (comma-separated value) format.

### 2.5. Identification of Volatile Coffee Green Bean

Identification of metabolites was done by comparing the mass spectra of each peak that appeared on the chromatogram with the data presented in the NIST 14 (National Institute of Science & Technology, 2014) database. Only the metabolites with a similarity index (SI) above 80% (considered a satisfactory match) were identified. To confirm the metabolite name, further identification was performed by matching the sample LRI values under the experimental condition with LRI values reported in published papers. The same value of LRI between samples compound and literature compound in combination with mass spectral information can be accepted and widely used for compound identification as described by [43].

### 2.6. Data Processing

The raw data from the SPME-GC-MS analysis included *m*/*z* values, retention times, and intensity. The data were tabulated and converted into a CSV format file. The data format was created in one folder and compressed into a single ZIP file (data compression). The data were processed and statistically analysed on the MetaboAnalyst 5.0 platform, which is freely available online at https://www.metaboanalyst.ca/MetaboAnalyst/ModuleView.xhtml. After the data were uploaded, data processing was performed, which included checking data integrity, handling missing values, data filtering, and data normalization. All data were Pareto scaled for normalization, and an unsupervised principal component analysis (PCA) was conducted to explore variations in the dataset.

### 2.7. Correlation of Temperature, Annual Rainfall, and Altitudes on Volatile Metabolite Profile

Annual rainfall in Bogor regions was significantly higher than that of Ciamis, Kuningan, Sumedang, and Tasikmalaya, that is, 4309.7 compared to 3226.6, 3226.6, 3104.0, 120.6 mm per year, respectively (Table 1). However, the average annual temperature in Bogor (26.4°C) region was significantly lower than that of Ciamis (28.7°C), Kuningan (28.7°C), Sumedang (28.7°C), and Tasikmalaya (30.8°C) regions (Table 1). The altitudes of the Robusta coffee in the five origins of robusta coffee plantation in West Java vary, 398.5 m at Tasikmalaya, 551 m at Sumedang, 607 at Ciamis, 657 at Kuningan, and 700 m at Bogor (Table 1). To reveal whether any relationship between temperature, annual rainfall, and altitudes with the relative concentration of discriminant volatile organic metabolites (VOMs) in Robusta green coffee beans, the Pearson correlation coefficient (*r*) was analysed.

### 2.8. Statistical Analysis

The dataset was subjected to multivariate statistical analysis, including PCA and partial least squares discriminant analysis (PLS-DA). PCA and PLS-DA are multivariate analyses that classify and differentiate samples based on the multidimensional data generated from the sample analysis technique used [44]. Additionally, variable importance in the projection (VIP) statistics from PLS-DA modeling was utilized to identify discriminant metabolites. VIPs > 1.5 were considered highly relevant for group discrimination [45]. Furthermore, hierarchical clustering analysis (HCA) of the entire dataset was conducted to reveal the similarities and differences between green coffee bean samples based on metabolite content. According to [46], the objective of heat map HCA is to separate samples into clusters, with the most similar objects combined to form a single cluster based on their similarities across multiple variables. Subsequently, the correlation between discriminant metabolites and climatic factors of sample origins, such as temperature, rainfall, and altitudes, was assessed using the Pearson correlation coefficient.

## 3. Results and Discussion

### 3.1. Profile of Volatile Metabolites in the Robusta Green Bean Coffee

In this study, SPME GC-MS detected 143 VOMs in single green coffee beans from five different regions. Identification was achieved by comparing their mass spectra with reference data stored in the NIST 14 database. Among these metabolites, 69 were confirmed by comparing their LRIs with values reported in published papers, as listed in Table 2. These metabolites were classified into various classes, including monounsaturated hydrocarbons, alcohols, monoterpenes, furans, alkanes, pyrazines, aldehydes, aromatic hydrocarbons, carboxylic acids, sesquiterpenes, ketones, pyrroles, terpenoids, phenols, glycols, fatty acids, indoles, lactones, oxacycles, phenolic compounds, and benzofurans. Notably, the dominant classes were aromatic hydrocarbon, aldehydes, alcohols, monoterpene, pyrazines, sesquiterpenes, carboxylic acids, and terpene. Similar results regarding the abundant content of alcohols and aldehydes in green coffee beans were also reported by [30].

The class of pyrazines reported was also found to be abundantly present in Robusta coffee beans, such as 2-methyl-pyrazine, 2,6-dimethylpyrazine, 2,5-dimethylpyrazine, ethylpyrazine, and 2-ethyl-6-methylpyrazine [72, 73]. Some of these compounds were also found in this study, including 2,5-dimethylpyrazine and 2-ethyl-6-methylpyrazine. Furthermore, several metabolites, such as acetic acid, 2.5-dimethyl-pyrazine, 2-ethyl-5-methyl-pyrazine, trimethyl-pyrazine, 3-ethyl-2.5-dimethyl-pyrazine, phenol, and furfurylpyrrole, detected in this study, were also found in coffee samples from the Philippines [19]. Additionally, [34] detected the presence of the same volatile compounds as those found in the coffee samples in this study, including acetic acid, 2.5-dimethyl-pyrazine, 2-ethyl-6-methyl-pyrazine, 2-ethyl-5-methyl-pyrazine, 3-ethyl-2.5-dimethyl-pyrazine, acetic acid, 2-ethyl-3.5-dimethyl-pyrazine, and phenol. From this comparison, it means that the content of volatile metabolites in Robusta green coffee beans is highly similar. This most likely occurs due to genetic similarity that governs metabolite production, leading to similar volatile metabolite profiles.

The data set generated from SPME GC-MS was subjected to PCA to assess the similarities and dissimilarity of the 143 volatile metabolites in the five samples of green bean coffee from five different regions in West Java. PCA is an unsupervised method that plays an important role for the discrimination and grouping of chemicals in a sample of food and medicine [74]. Through PCA, a large data set can be summarized; thus, the diversity in the data can be seen, and the large data set can be grouped.

**Figure 2:**
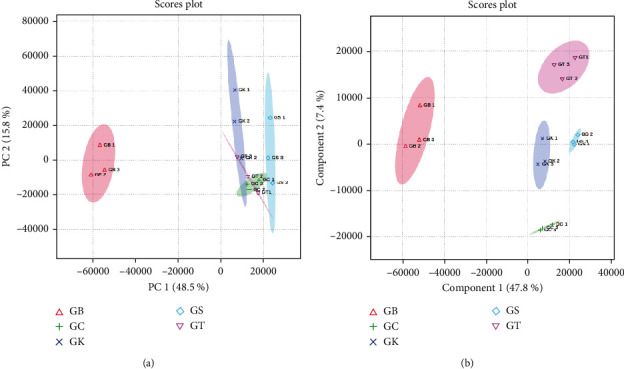
(a) 2D score plot PCA and (b) 2D score plot PLS-DA of the metabolites obtained in green beans from five different geographical origins in West Java, that is, GB (green beans from Bogor), GC (green beans from Ciamis), GK (green beans from Kuningan), GS (green beans from Sumedang), and GT (green beans from Tasikmalaya).

The results of PCA of the entire metabolites showed that there was a diversity of volatile metabolite profiles between each coffee sample from the five regions, that is, 48.5% based on PC1 and 15.8% based on PC2; thus, the combination of PC1 and PC2 could explain a cumulative of 64.3% metabolite variability among the green bean Robusta samples from five regions in West Java, as indicated in PCA score plots (Figure 2(a)). The score plots of the PCA of the five coffee samples showed that the position of the coffee samples from Bogor was distinct and separated from other sample groups. This means that the metabolite profiles of coffee from Bogor were clearly different from the other four green bean samples (samples from Kuningan, Sumedang, Ciamis, and Tasikmalaya). Meanwhile, green beans of coffee samples from Ciamis, Tasikmalaya, Kuningan, and Sumedang seem to gather at adjacent points. This is interesting as the Bogor region is geographically located far from Ciamis, Tasikmalaya, Kuningan, and Sumedang, while Ciamis, Tasikmalaya, Kuningan, and Sumedang are geographically close to each other (Figure 1). Therefore, it is notable that samples from those regions geographically close to each other have almost similar metabolite profiles. This result was in agreement with previous findings reported by [58].

Pevious study reported that variability of single beans coffee volatile compound derived from SPME-GC-MS analysis coupled with linear discrimination analysis (LDA) and multiple layer perception (MLP) could classify single–roasted coffee bean samples according to their countries of origin, that is, Brazil, Colombia, Costa Rica, Ethiopia, Guatemala, Honduras, India, Kenya, Mexico, Nicaragua, Rwanda, Uganda, and Vietnam [37]. Moreover, [19] reported that through PCA of the SPME GC-MS–derived data set and its multivariate analysis gave rise to metabolite profiles of Robusta and Arabica coffee samples and allowed the discrimination between Robusta and Arabica based on the profile of the metabolites [19]. Similarly, [20] reported that the SPME GC-MS data set of volatile compounds coupled with multivariate statistical analysis could profile and discriminate 47 ground coffee samples [20]. Thus, the combination of metabolite analysis using SPME-GC-MS and multivariate analysis is effective in distinguishing coffee samples. However, the present study has some limitations, and we consider the extraction results of our robusta coffee bean samples using SPME coupled with GC-MS analysis reliable. Nevertheless, there is a possibility that some metabolites may not be detected by these methods, as SPME is most effective for volatile and semivolatile compounds. Nonvolatile or less volatile compounds may not be efficiently extracted, leading to incomplete profiling of metabolites. Therefore, for future work, it is necessary to verify the results to strengthen the perception of these findings by using different analytical techniques, such as sample extraction techniques using solvents followed by derivatization for detection by GC-MS.

### 3.2. Discriminant Metabolites Between Different Regions of Coffee Plantations

From the metabolite profile data, the analysis was continued to PLS-DA to reveal the metabolic difference among the samples. The results of the PLS-DA with a total variance of 56.0% (PC1 = 48.6% and PC2 = 7.4%) could separate the samples more sharply and could classify the green beans of the Robusta coffee based on the region from which the samples were cultivated (Figure 2(b). This happens because PLS-DA can further analyse and process data by looking at the groups of input data. In addition, through this approach, potential discriminants among samples can be determined [75].

Through the PLS-DA analysis, the value of VIP can be determined. The VIP value is a determinant of the confidence value of the importance of a variable. In this work, nine metabolites were generated with VIP scores > 1.5 which include benzaldehyde, isovaleric acid, toluene, diisobutyl succinate, 1-heptene, 4-dodecene, caffeine, acetic acid, and methyl benzoate. The nine metabolites were candidate discriminant metabolites (biomarkers) from the total peak that was analysed by the MetaboAnalyst system.

**Figure 3:**
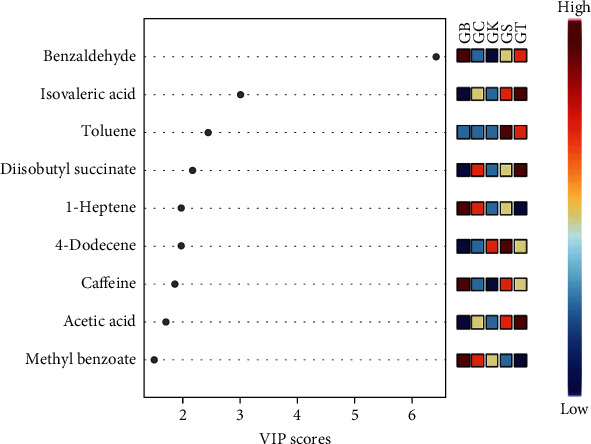
Variable importance in projection (VIP) scores PLS-DA of Robusta green bean coffee samples from five different geographical origins: GB (green beans from Bogor), GC (green beans from Ciamis), GK (green beans from Kuningan), GS (green beans from Sumedang), and GT (green beans from Tasikmalaya).

The colour gradient on the VIP chart shows the relative concentrations of the discriminant of the analysed sample groups, red indicates a higher relative concentration, and blue indicates a lower relative concentration of metabolites (Figure 3). Among the nine peak compounds, seven of them were identified according to NIST 14 mass spectra and further confirmed by comparison of their LRI value with the similar value of LRIs in the published paper, as presented in Table 3. However, two compounds, 4-dodecene and caffeine, were only tentatively identified based on NIST 14 mass spectra. The method of metabolite identification by using a combination of the mass spectra from GC-MS and LRIs is widely accepted and routinely used to confirm the identity of compounds [43].

**Figure 4:**
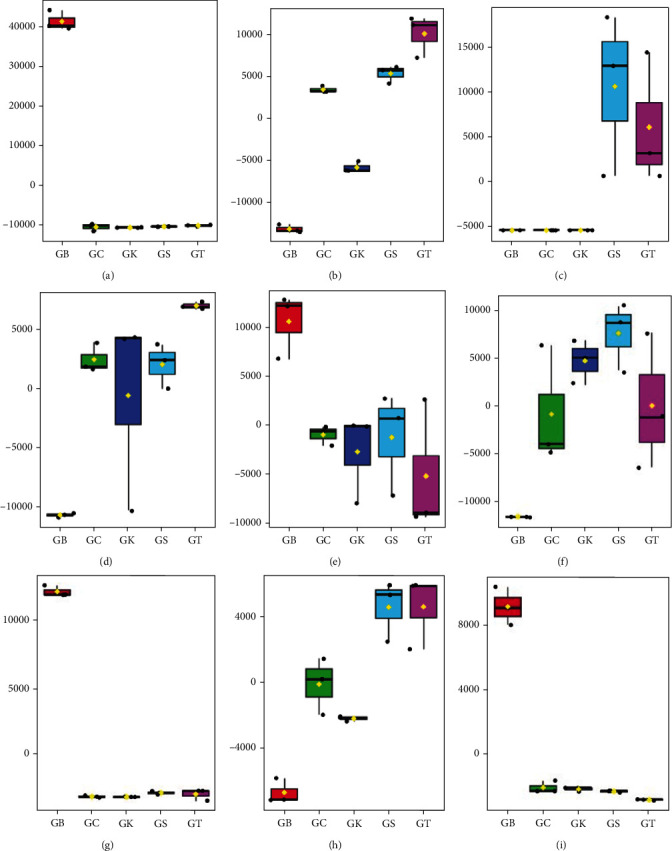
Boxplot relative concentrations of the main discriminant metabolites, (a) benzaldehyde, (b) isovaleric acid, (c) toluene, (d) diisobutyl succinate, (e)1-heptene, (f) 4-dodecene, (g) caffeine, (h) acetic acid, and (i) methyl benzoate.

The comparison of the nine candidates of discriminant metabolites (biomarkers) among the five samples of Robusta green bean coffee indicated that the relative concentration of benzaldehyde, 1-heptene, caffeine, and methyl benzoate was markedly higher in Robusta green bean from Bogor than that of Ciamis, Kuningan, Sumedang, and Tasikmalaya as represented on box plot metabolites (Figures 4(a), 4(e), 4(g), and 4(i)), while isovaleric acid, diisobutyl succinate, 4-dodecene, and acetic acid were found in relatively higher concentrations in green bean coffee from Ciamis, Kuningan, Sumedang, and Tasikmalaya compared to that from Bogor (Figures 4(b), 4(d), 4(f), and 4(h)). Meanwhile, toluene was found in relatively higher concentrations in the green beans from Sumedang and Tasikmalaya compared to the green beans from Bogor, Ciamis, and Kuningan (Figure 4(c)). These findings suggest that the relatively higher concentrations of benzaldehyde, 1-heptene, caffeine, and methyl benzoate may serve as discriminant VOMs to distinguish Bogor Robusta green coffee beans, while isovaleric acid, diisobutyl succinate, 4-dodecene, acetic acid, and toluene could serve as a volatile metabolites marker for Robusta green beans from Ciamis, Kuningan, Sumedang, and Tasikmalaya. It was seen that each region's beans can be identified by their specific volatile metabolite profile. Kuningan beans (GK) show relatively higher levels of diisobutyl succinate and 4-dodecene, while Ciamis beans (GC) have relatively higher isovaleric acid, diisobutyl succinate, and 4-dodecene. The profiles of beans from Sumedang (GS) and Tasikmalaya (GT) were similar, with relatively higher concentrations of isovaleric acid, diisobutyl succinate, 4-dodecene, toluene, and acetic acid.

The differences in metabolite concentrations in green coffee beans were suspected to be caused by the environmental factors where the coffee plants were grown. In regions with relatively lower temperatures and higher rainfall, such as in Bogor, the accumulation of benzaldehyde, 1-heptene, caffeine, and methyl benzoate was stimulated. Conversely, higher temperatures and relatively lower rainfall such as in Ciamis, Kuningan, Sumedang, and Tasikmalaya induce an increase in the concentrations of isovaleric acid, diisobutyl succinate, 4-dodecene, acetic acid, and toluene. This indicates that there is a close relationship between the environmental conditions where the coffee plant grows and the cellular metabolic processes occurring within the coffee beans, leading to the synthesis or degradation of certain metabolites. This finding is similar to [33], who stated that the growing environment has a strong effect on the biochemical composition of the coffee plant.

Each discriminant metabolite has a role in the flavor and aroma of coffee, potentially giving rise to a distinctive odor to distinguish Robusta coffee from the Bogor cluster from those of the Ciamis, Kuningan, Sumedang, and Tasikmalaya clusters, as shown in Table 3. As the presence of these metabolites is believed to impact sensory quality, Robusta coffee from the Bogor cluster, with higher concentrations of benzaldehyde, 1-heptene, caffeine, and methyl benzoate, could be assumed to have a strong aroma, fragrance, and flavor, possessing more bitter and fruity sensory attributes. In contrast, coffee from Ciamis, Kuningan, Sumedang, and Tasikmalaya exhibits sensory attributes of cheesy, fruity, nutty, and sour, due to higher concentrations of isovaleric acid, diisobutyl succinate, 4-dodecene, and acetic acid.

### 3.3. Hierarchical Clustering

**Figure 5:**
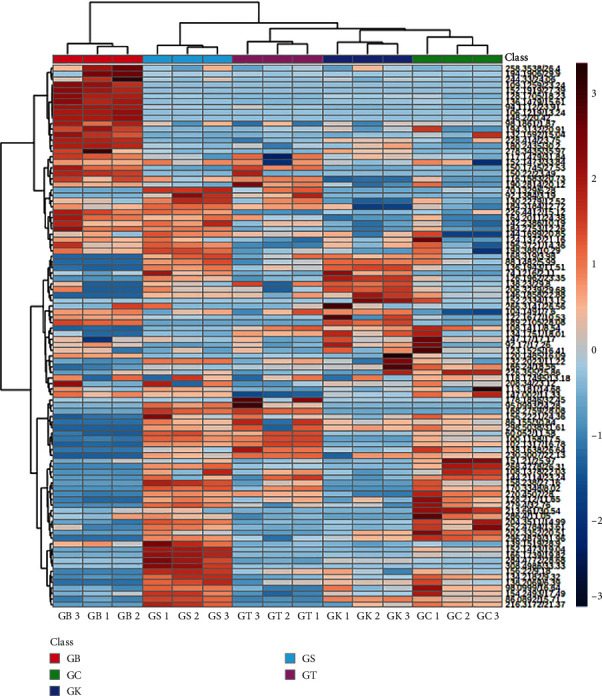
Heat map hierarchical clustering of the volatile metabolites data set of green bean coffee from five different geographical origins: GB (green beans from Bogor), GC (green beans from Ciamis), GK (green beans from Kuningan), GS (green beans from Sumedang), and GT (green beans from Tasikmalaya). Each column represents a different sample, and the row represents the different metabolites. Data set is comprised of 5 types of samples, each type of sample was prepared with 3 replications of biological material.

Here, heat maps are displayed and accompanied by a dendrogram. The results of the heat map cluster analysis on the metabolite profiles of each coffee sample from five different regions showed that Bogor green coffee (GB) was in a different dendrogram branch (clade) from the GC, GT, GK, and GS groups (Figure 5). This means that there were significant differences in volatile metabolite profiles of Bogor robusta coffee compared to that of Ciamis green coffee (GC), green coffee Tasikmalaya (GT), green coffee Kuningan (GK), and green coffee Sumedang (GS). Therefore, Bogor green coffee (GB) is in a separate cluster. On the other hand, GC, GT, GK, and GS were included in another cluster, with GT, GK, GC, and GS showing more similar profiles, being included in the same subcluster together. Therefore, overall coffee samples were classified into two main clusters, Cluster 1 (Robusta green beans from Bogor) and Cluster 2 (Robusta green beans from Ciamis, Kuningan, Sumedang, and Tasikmalaya). Apparently, this clustering result was in accordance with the result of the PCA, in which the profile metabolite of green beans from Bogor differs from that of Ciamis, Kuningan, Sumedang, and Tasikmalaya, while the profile metabolite of green beans from Ciamis, Kuningan, Sumedang, and Tasikmalaya showed similarity. This result indicates that there is variation within the same genotype of Robusta coffee which is cultivated in different geographical origins.

The presence of variations in the chemical composition of the Robusta green bean coffee at several different locations probably was related to environmental factors where the coffee tree was grown. According to previous different study, the biochemical composition in green bean coffee depends on plant genetic differences [8], geographic origin [14], seasonal variation of harvested coffee [13], pre and postharvest processing of the beans, that is, wet or dry-processing [15], the presence of defective seeds [16], and fruit maturity [17].

In the present study, all samples analysed had the same genotype, same harvest time, same method for picking the cherries, same fruit ripeness, and same postharvest processing that was through dry processing. The different factor among the samples analysed was the geographical origin from which the samples were collected. Most probably, the differences in volatile metabolite profiles in Cluster 1 (robusta green bean from Bogor) with Cluster 2 (robusta green bean from Ciamis, Kuningan, Sumedang, and Tasikmalaya) were associated with the environmental conditions of the region where Robusta coffee was cultivated. Among the environmental factors, this study highlighted rainfall and temperature for investigation because the levels of rainfall and temperature in the five coffee–producing areas were significantly different (Table 1).

### 3.4. Correlation of Temperature, Annual Rainfall, and Altitudes on Volatile Metabolite Profile

Pearson's correlation analysis between temperature and the nine discriminant metabolites (Table 4) showed that temperature had a strong positive correlation with isovaleric acid (*r* = 0.8), diisobutyl succinate (*r* = 0.8), acetic acid (*r* = 0.76), and moderately with toluene (*r* = 0.5) and 4-dodecene (*r* = 0.4). Additionally, a strong negative correlation of temperature was seen with 1-heptene (*r* = −0.78), methyl benzoate (*r* = −0.63), caffeine (*r* = −0.59), and weakly with benzaldehyde (*r* = −0.23). These correlations indicate that higher temperatures were associated with an increase in the contents of isovaleric acid, diisobutyl succinate, acetic acid, toluene, and 4-dodecene, but a decrease in 1-heptane, methyl benzoate, caffeine, and benzaldehyde.

Another study indicated that when the temperature rises, the volatile compounds in green beans of *Coffea arabica* such as ethanol, benzene ethanol, hexanoic acid, pentanoic acid, butyrolactone, phenylacetaldehyde, and dodecene increase, whereas 2,3-butanediol decreases [41]. Apparently, in this study, there was an increase in 4-dodecene as temperatures rose; however, benzaldehyde decreased when the temperature rose. Those were in line with the results of the study [41].

The temperature in Bogor was significantly lower than in other areas. Under these conditions, certain metabolites such as benzaldehyde, caffeine, 1-heptene, and methyl benzoate accumulated more prominently compared to those in other areas. This finding is consistent with studies reporting that caffeine accumulation is influenced by environmental factors [77]. Lower temperatures delay the ripening of coffee by about a month and significantly alter the biochemical composition during bean development. Consequently, the accumulation of caffeine tends to be higher [16].

Pearson's correlation analysis of rainfall (Table 4) showed a strong positive correlation with caffeine (*r* = 0.76), methyl benzoate (*r* = 0.79), and moderately with benzaldehyde (*r* = 0.43). However, it exhibited a strong negative correlation with 1-heptene (*r* = −0.88), isovaleric acid (*r* = −0.88), diisobutyl succinate (*r* = −0.94), acetic acid (*r* = −0.86), and moderate negative correlation with 4-dodecene (*r* = −0.52) and toluene (*r* = −0.64) (Table 4). These results indicate that an increase in rainfall leads to an increase in benzaldehyde, caffeine, and methyl benzoate, but a decrease in 1-heptene, isovaleric acid, diisobutyl succinate, acetic acid, and toluene. Another study reported that 2,3-butanediol and 4-dodecene were highly correlated with soil water availability [41]. It appears that there is a difference in the correlation of 4-dodecene with the level of rainfall, which may be due to the different types of coffee used—Robusta coffee in this study and Arabica coffee in the study by [41].

Pearson's correlation analysis of altitudes indicated that among the discriminant metabolites, benzaldehyde, diisobutyl succinate, caffeine, and acetic acid demonstrated weak correlations with altitude, with *r* values of −0.181, 0.257, 0.095, and 0.008, respectively. Meanwhile, methyl benzoate showed a strong positive correlation (*r* = 0.70), and 1-heptene showed a moderately positive correlation (*r* = 0.57). Isovaleric acid, toluene, and 4-dodecene showed strong negative correlations with *r* values of −0.66, −0.74, and −0.94, respectively (Table 4). This indicates that only methyl benzoate, 1-heptene, isovaleric acid, toluene, and 4-dodecene have strong correlations with altitude (either positive or negative). However, most of the VOMs from Robusta green coffee beans grown at altitudes ranging from 398.5 to 700 m demonstrated a weak correlation with altitude. Correspondingly, [30] discovered that the volatile metabolites in Arabica green coffee beans grown at different altitudes in Ethiopia, ranging from 1515 to 2220 m, also showed a weak correlation with the altitude of the coffee plants.

Overall, from the correlation analysis of volatile metabolites with temperature, annual rainfall, and altitudes, it was revealed that the increase in temperatures was associated with an increase in the contents of isovaleric acid, diisobutyl succinate, acetic acid, toluene, and 4-dodecene, but a decrease in 1-heptene, methyl benzoate, caffeine, and benzaldehyde corresponding to the volatile metabolites profile of GK, GC, GS, and CT. The increase in rainfall leads to an increase in benzaldehyde, caffeine, and methyl benzoate, but a decrease in 1-heptene, isovaleric acid, diisobutyl succinate, acetic acid, and toluene corresponding to the volatile metabolites profile of GB; the relatively high altitude such as in Bogor correlate with the relatively high concentration of methyl benzoate and 1-heptene, but in low concentration in isovaleric acid, toluene, and 4-dodecene. This condition in Bogor is related to the phenomenon that as altitude increases, the temperature generally decreases. The correlation tests between these three environmental factors and the discriminant metabolites gave consistent results with respect to temperature, rainfall, and altitude.

In relation to the current issue of climate change due to global warming, the results of this study indicate that temperature is a crucial factor in the metabolism of coffee plants. The 2°C temperature difference between Robusta coffee cultivation sites in Bogor and other regions in West Java, such as Kuningan, Ciamis, and Sumedang, as well as the 4°C difference with Tasikmalaya, has led to variations in the relative concentrations of several key discriminants. It has also been demonstrated that the amount of rainfall affects the relative concentrations of several key metabolites. In the Bogor region, which has relatively high rainfall, the concentrations of 1-heptene, methyl benzoate, caffeine, and benzaldehyde are higher compared to those in areas with significantly lower rainfall. Therefore, in the future, if there is an increase in temperatures and variations in rainfall as a result of climate change, it is suspected that this will alter the volatile compound profile of green coffee, ultimately affecting the quality of the green coffee beans.

This could negatively impact the consistency of coffee quality and create challenges for coffee farmers to maintain the desired chemical profile from year to year. Additionally, it may lead to shifts in the optimal cultivation areas for coffee or force coffee plants to grow under less ideal conditions, which could decrease the chemical quality of the coffee beans. Thus, adaptation strategies are needed, particularly for coffee farmers in adapting their cultivation practices to mitigate the negative impacts of climate change, which can reduce both the production and quality of the coffee beans produced.

These findings are interesting; however, this study is limited to the robusta coffee regions in West Java during a specific harvest season, and thus, the results cannot be generalized. Further research seems necessary using a larger sample size across multiple harvest seasons. In addition, this study is limited by the absence of a discussion on other environmental factors such as soil conditions, light intensity, shading, and farming practices, which can also influence the chemical composition of green coffee beans. Therefore, additional environmental factors that might affect the metabolite profile of Robusta coffee, including soil condition, light intensity, shading, and farming practices, need to be tested. Moreover, although green coffee beans can influence the final coffee cup profile through the availability of various precursor metabolites that will form after roasting, since the cup profile is also determined by the roasting temperature, it is advisable to also conduct volatile metabolite profiling on roasted coffee to better assess the quality directly experienced by coffee drinkers.

## 4. Conclusions

The combination of metabolite analysis technologies (e.g., SPME-GC-M) and multivariate analysis is effective for distinguishing the geographical origin of Robusta coffee in West Java, based on their volatile metabolite profiles. Each region produces Robusta coffee with distinct volatile compounds. For example, the metabolite profile of Bogor Robusta green beans is characterized by higher levels of benzaldehyde, 1-heptene, caffeine, and methyl benzoate; Kuningan beans by higher levels of diisobutyl succinate and 4-dodecene; Ciamis beans by higher levels of isovaleric acid, diisobutyl succinate, and 4-dodecene, while Sumedang and Tasikmalaya beans are nearly indistinguishable, both containing higher levels of isovaleric acid, diisobutyl succinate, 4-dodecene, toluene, and acetic acid. The distinct profile of green coffee beans from Bogor is associated with significantly higher rainfall and lower temperatures, highlighting the important role of microclimate factors in shaping these metabolite profiles.

This study offers important insights into the variations in metabolites of single-variety Robusta green beans based on their cultivation locations in West Java, which may help confirm the authenticity of coffee according to its geographical origin. Moreover, the findings underscore how temperature and rainfall influence the volatile metabolite profile, leading to variations in key compounds that may affect coffee quality. In relation to climate change, shifts in temperature and rainfall could present challenges for maintaining consistent coffee quality and may require adaptation strategies for farmers, such as relocating coffee cultivation to new areas.

Although this study effectively profiles volatile and semivolatile metabolites using SPME, it has limitations, including the potential for undetected metabolites and a focus on specific Robusta coffee–growing areas in West Java during a single harvest period. Thus, these results cannot be generalized. Future research should involve larger sample sizes and multiple harvest seasons and consider additional environmental factors such as soil conditions, light intensity, shading, and farming practices. It is also recommended to profile volatile metabolites in roasted coffee, as the roasting process significantly impacts the final coffee quality perceived by consumers.

## Figures and Tables

**Table 1:** Sampling sites of Robusta green coffee bean with their coordinates, annual rainfall, elevation, annual temperature, and harvest season.

**No.**	**Sampling site**	**Coordinates**	**Average annual rainfall (mm/year)**	**Altitude (masl)**	**Average annual temp. (°C)**	**Harvest season**
1	Bogor	S/South Latitude 06°38.357⁣^″^ E/East Longitude 107°07⁣′239⁣^″^	4309. 7^a^	700	26.4^x^	2021
2	Ciamis	S/South Latitude 07°08.076⁣^″^ E/East Longitude 108°23⁣′365⁣^″^	3226.6^b^	607	27.8^y^	2021
3	Kuningan	S/South Latitude 01^o^51.605⁣^″^ E/East Longitude 108°28⁣′006⁣^″^	3226.6^b^	657.5	27.8^y^	2021
4	Sumedang	S/South Latitude 06°53.430⁣^″^ E/East Longitude 107°59⁣′418⁣^″^	3104.0^b^	551	27.8^y^	2021
5	Tasikmalaya	S/South Latitude. 06°38.438⁣^″^ E/East Longitude. 107°08⁣′014⁣^″^	120.6^c^	398.5	30.8^z^	2021

*Note:* Coordinates and altitudes were measured using Garmin GPSMap 78; annual rainfall and temperature were obtained from Statistics Indonesia West Java Province agency (https://jabar.bps.go.id/indicator/151/430/1/-curah-hujan-di-stasiun-pengamatan-klimatologi); values with different super script letters in the Columns 4 and 6 indicate significant differences with a *p* value < 0.05.

Abbreviation: masl, meter above sea level.

**Table 2:** List of identified volatile metabolites in the single robusta green bean coffee from five different regions in West Java.

**Metabolites**	**RT**	**LRI exp.**	**LRI lit.**	**Class**
1-Heptene	1.824	992.7	961	Monounsaturated hydrocarbons
1-Butanol	2.0856	921.08	955	Alcohols
Toluene	3.1855	1046.65	1049	Aromatic hydrocarbons
Limonene	5.7423	1204.34	1206	Monoterpene
Isoamyl alcohol	5.992	1214.92	1204	Alcohol
2-Amylfuran	6.3844	1231.543	1232	Furan
*β*-Ocimene	6.8007	1249.178	1250	Monoterpene
Styrene	6.9613	1255.98	1247	Aromatic hydrocarbons
Cyclooctatetraene	7.6926	1286.96	1255	Cyclic hydrocarbons
2.5-Dimethyl- pyrazine	8.537	1321.64	1320	Pyrazines
*α*-Methylstyrene	8.657	1326.48	1371	Aromatic hydrocarbons
p-Cymene	9.3159	1353.05	1437	Aromatic hydrocarbons
Neoallo-ocimene	9.543	1362.214	1366.5	Monoterpene
2-Ethyl-6-methyl- pyrazine.	10.071	1383.5	1394	Pyrazines
Nonanal	10.184	1388.06	1392	Aldehydes
2-Ethyl-5-methyl- pyrazine.	10.19	1388.31	1401	Pyrazines
Tetradecane	10.291	1392.38	1400	Alkanes
Trimethyl- pyrazine.	10.5289	1401.91	1414	Pyrazines
p-Cymenene	11.2304	1429.286	1437	Monoterpene
1.3-Dichloro-benzene	11.3316	1433.23	1425	Aromatic hydrocarbons
3-Ethyl-2.5-dimethyl- Pyrazine	11.51	1440.19	1444	Pyrazines
Acetic acid	11.5754	1442.75	1450	Carboxylic acids
1-Octen-3-ol	11.6467	1445.53	1590	Alcohols
2-Ethyl-3.5-dimethyl pyrazine	11.9143	1455.97	1444	Pyrazines
2-Ethylhexyl acrylate	12.4078	1475.23	1496	Acrylic acids
Benzaldehyde	13.2402	1508.23	1513	Aldehydes
Vanillin. propyl ether	13.9001	1535.698	1550	Phenolic compounds
Linalool	13.9834	1539.166	1543	Monoterpenes
Heptane. 3.3.5-trimethyl-	14.1321	1545.35	1487	Alkane
Longifolene	14.4413	1558.23	1568	Sesquiterpenes
Thiazole. 4.5-dimethyl-	14.584	1564.17	1447	Thiazoles
*β*-Elemene	14.8813	1576.55	1590	Sesquiterpenes
2-Methyl- benzofuran	15.024	1582.5	1482	Thiazoles
Hexadecane	15.1191	1586.4	1596	Alkanes
Methyl benzoate	15.6067	1607.05	1605	Aromatic esters
Butyrolactone	15.665	1609.6	1628	Lactones
Benzeneacetaldehyde	16.017	1624.9	1623	Aldehydes
4-Methylbenzaldehyde	16.169	1631.56	1538	Aldehydes
Acetophenone	16.2548	1635.29	1643	Aromatic ketones
2-Acetyl-1-methylpyrrole	16.402	1641.7	1663	Pyrroles
3-Furanmethanol	16.6294	1651.6	1659	Furan
Isovaleric acid	16.784	1658.35	1665	Carboxylic acids
Dimethyl- silanediol	17.2538	1678.8	1638	Silanes
*cis*-*α*-Bisabolene	18.0505	1714.04	1740	Sesquiterpenes
Naphthalene	18.223	1721.84	1751	Aromatic hydrocarbons
Methyl salicylate	19.0494	1759.2	1804	Aromatic esters
*β*-Damascenone	20.1138	1807.6	1839	Terpenes
2-Furfurylpyrrole	20.2208	1812.67	1806	Pyrroles
5-Ethyl-2-octen-4-one	20.5003	1825.84	1649	Ketones
Calamenene	20.5061	1826.113	1832	Sesquiterpenes
1-Methylnaphthalene	20.649	1832.84	1735.6	Aromatic hydrocarbons
Caproic acid	20.6786	1834.24	1843	Carboxylic acid
Dihydropseudoionone	20.8392	1841.8	1885	Terpenes
2-Methoxy-phenol	20.91	1845.14	1877	Phenol
1.3-Pentanediol,2.2.4-trimethyl-diisobutyrate	21.336	1865.2	1875	Glycols
Benzyl alcohol	21.3446	1865.6	1869	Alcohols
Diisobutyl succinate	21.9511	1894.2	1686	Fatty acid
Phenylethyl alcohol	22.1235	1902.36	1933	Alcohols
Dicyclopentenyl alcohol	23.5089	1969.12	1701	Alcohols
2-Pentadecanol	23.7646	1981.5	1770	Acohols
Phenol	23.9073	1988.3	1956	Aromatic hydrocarbons
Glutaric acid. di(isobutyl) ester	24.044	1994.9	1958	Esters
*γ*-Nonalactone	24.3651	2013.5	2023	Oxacycle
Isobutyl adipate	26.3987	2134.5	2140	Esters
4-Vinylguaiacol	27.5224	2194.08	2134	Phenolic compounds
Dihydroactinidiolide	30.1922	2341.5	2348	Lactones
Dihydrobenzofuran	31.0187	2388.3	2358	Benzofurans
Indole	31.8392	2436.35	2435	Indoles

*Note:* LRI exp.: linear retention indices in this experiment; LRI lit.: the LRI scores are taken from the following literatures [47–71].

Abbreviation: RT: retention time.

**Table 3:** Association of the discriminant compounds (VIP score > 1.5) in the green coffee and their sensory attributes.

**No**	**Compound name**	**VIP score**	**L** **R** **I**_exp_	**L** **R** **I**_**l** **i** **t**_	**Sensory attributes**
1	Benzaldehyde	6.4	1508.23	1513	Aroma almond
2	Isovaleric acid	3.2	1658.35	1665	Cheesy
3	Toluene	2.7	1046.65	1049	Sharp or sweet
4	Diisobutyl succinate	2.2	1894.2	1686	Fruity
5	4-Dodecene	2.1	1132.52	.	Nutty
6	1-Heptene	2.1	992.7	961	Fragrance and flavour
7	Acetic acid	1.7	1442.75	1450	Sour
8	Caffeine	2	2328.7	.	Bitter
9	Methyl benzoate	1.5	1607.05	1605	Fruity

*Note:* Description of sensory attributes of the related compounds are taken from PubChem, National Library of Medicine [76].

**Table 4:** Correlation coefficient between the temperature, rainfall, and altitudes with nine discriminant metabolites.

**Pearson correlation coefficient (** **r** **)**									
**Climatic factors**	**Benzaldehyde**	**Isovaleric acid**	**Toluene**	**Diisobutyl succinate**	**1-Heptene**	**4-Dodecene**	**Caffeine**	**Acetic acid**	**Methyl benzoate**
Annual temp.	−0.227	0.805	0.53	0.84	−0.78	0.389	−0.586	0.763	−0.634
Annual rainfall	0.432	−0.889	−0.593	−0.94	−0.889	−0.517	0.758	−0.862	0.796
Altitudes	−0.181	−0.661	−0.74	0.256	0.569	−0.944	0.0947	0.008	0.704

*Note:* The *p* value for each correlation coefficient is < 0.5.

## Data Availability

All the data relevant to the research can be found in the manuscript. Further information is available from the corresponding author upon request.
